# Ultrafast helicity control of surface currents in topological insulators with near-unity fidelity

**DOI:** 10.1038/ncomms7617

**Published:** 2015-03-26

**Authors:** Christoph Kastl, Christoph Karnetzky, Helmut Karl, Alexander W. Holleitner

**Affiliations:** 1 Walter Schottky Institut and Physik-Department, Technische Universität München, Am Coulombwall 4a, 85748 Garching, Germany; 2 Nanosystems Initiative Munich (NIM), Schellingstr. 4, 80799 München, Germany; 3 Institute of Physics, University of Augsburg, 86135 Augsburg, Germany

## Abstract

In recent years, a class of solid-state materials, called three-dimensional topological insulators, has emerged. In the bulk, a topological insulator behaves like an ordinary insulator with a band gap. At the surface, conducting gapless states exist showing remarkable properties such as helical Dirac dispersion and suppression of backscattering of spin-polarized charge carriers. The characterization and control of the surface states via transport experiments is often hindered by residual bulk contributions. Here we show that surface currents in Bi_2_Se_3_ can be controlled by circularly polarized light on a picosecond timescale with a fidelity near unity even at room temperature. We reveal the temporal separation of such ultrafast helicity-dependent surface currents from photo-induced thermoelectric and drift currents in the bulk. Our results uncover the functionality of ultrafast optoelectronic devices based on surface currents in topological insulators.

## Abstract (web summary)

Bulk contributions to transport measurements often inhibit the study of the surface states of topological insulators. Here, Kastl *et al*. demonstrate high-fidelity helicity-dependent photocurrents in the surface states of Bi_2_Se_3_, controlled via circularly polarized light with a picosecond time-resolution.

## Graphical abstract

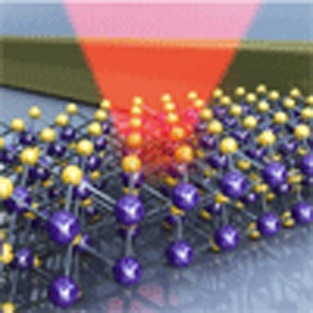

Layered materials, such as Bi_2_Te_3_ (refs 1, 2), Sb (ref. 3), Bi_2_Se_3_ (refs 2, 4, 5, 6), Bi_2_Te_2_Se (ref. 7) and (Bi_1-x_Sb_x_)_2_Te_3_ (ref. 8), are important narrow-bandgap semiconductors for tunable, high-performance infrared detectors and thermoelectric applications^9^. They have been demonstrated to be reference three-dimensional topological insulators^10^,^11^,^12^ exhibiting exceptional transport mobilities^1^,^2^,^3^,^4^,^5^,^6^. The latter suggests a reduced energy consumption that is very attractive for semiconductor devices in high-speed communication applications. In this respect, it is very advantageous that the helical surface states in topological insulators can be addressed by polarized light^2^,^9^,^13^,^14^,^15^,^16^. Particularly, the circular photogalvanic effect results from a helicity-dependent asymmetric optical excitation of spin-split surface states in momentum space, allowing the generation and control of spin-polarized surface currents in topological insulators by circularly polarized light^17^,^18^,^19^.

Here we demonstrate that such surface currents can be accessed and read-out independently of the bulk photocurrents on a picosecond timescale with near-unity fidelity. Therefore, our results open the avenue for a high-speed transmission of information based on topological insulators. Furthermore, our experiments address the connection between the non-equilibrium currents of hot electrons directly after the laser excitation and the time-averaged thermoelectric currents in these materials. A time-of-flight analysis of the photogenerated hot electrons yields a speed that is consistent with the group velocity at the Fermi-energy of the Bi_2_Se_3_.

## Results

### Time-resolved and time-integrated photocurrent spectroscopy

**Figure 1:**
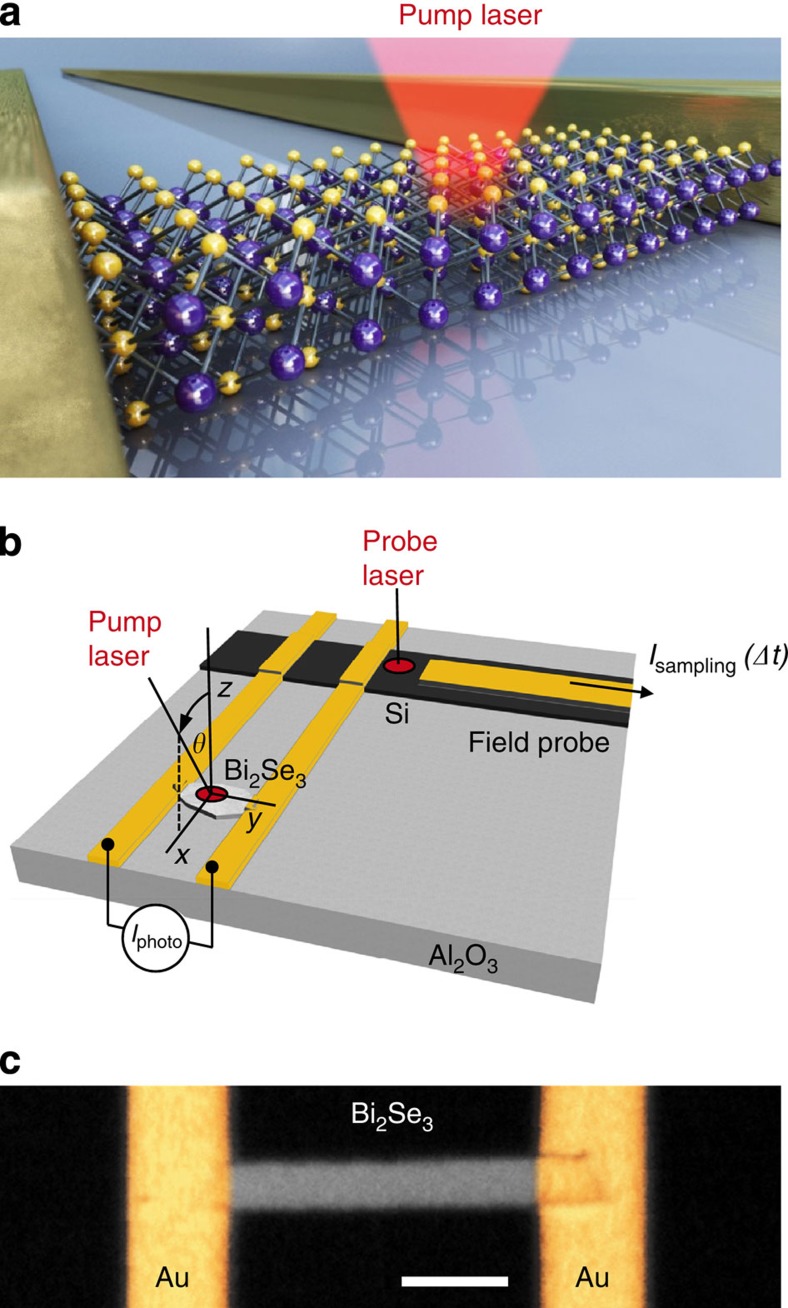
Ultrafast photodetector based on topological insulators. (**a**) Circularly polarized photons of the pump pulse control surface currents in Bi_2_Se_3_. (**b**) The time-resolved photocurrent *I*_sampling_ is read-out by a silicon-based Auston-switch triggered by a probe pulse at a time-delay Δ*t*. The time-integrated photocurrent *I*_photo_ is measured between the electronic contacts, which form two co-planar striplines. (**c**) Optical microscope image of a Bi_2_Se_3_ film with 75 nm thickness, contacted by two Au striplines. Scale bar, 5 μm.

The investigated n-type Bi_2_Se_3_ films are embedded in a metal-topological insulator-metal photodetector geometry (Fig. 1a). We characterize the ultrafast photocurrents by an on-chip, time-domain THz photocurrent spectroscopy with a picosecond time-resolution (Fig. 1b and Methods)^20^,^21^,^22^. We excite the Bi_2_Se_3_ films with a circularly polarized pump pulse under an oblique angle *θ* (Fig. 1b). Such helical photons excite spins asymmetrically in *k*-space owing to angular momentum selection rules in Bi_2_Se_3_ (refs 9, 23). In turn, a net out-of-equilibrium spin polarization is acquired in the Dirac cone. After photoexcitation, spin and charge degrees of freedom relax on different timescales in the bulk and surface states of Bi_2_Se_3_ (ref. 24). For the surface states, spin depolarization, intraband cooling via surface electron-phonon scattering and interband electron-hole recombination occur on a sub-picosecond to picosecond timescale^24^,^25^,^26^.

**Figure 2:**
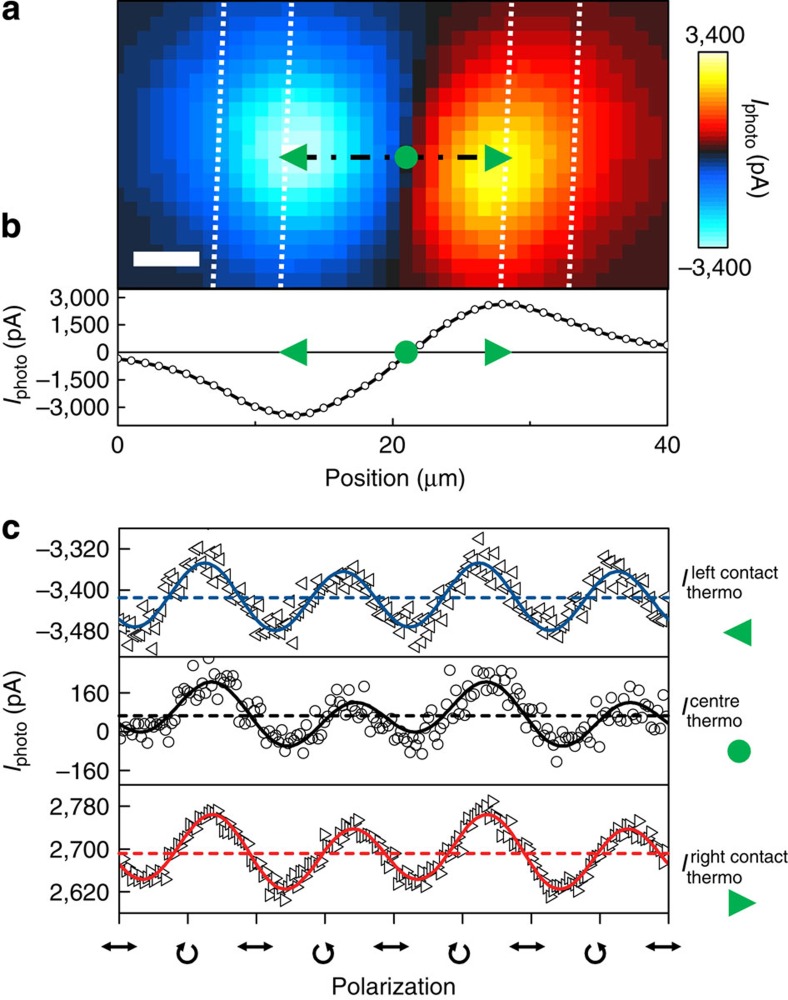
Time-averaged photocurrents in Bi_2_Se_3_. (**a**) Photocurrent map of *I*_photo_ in colour scale. Dotted lines indicate the position of the striplines. Scale bar, 5 μm. (**b**) Line scan along the black dashed dotted line in (**a**) with a positive (rightward triangle) and a negative peak (leftward triangle) close to the striplines. Circle denotes position of zero signal of *I*_photo_. (**c**) Polarization dependence of *I*_photo_. Positions are denoted in (**a**,**b**). The symbol denotes linearly polarized (along the *x*-axis), circularly right-handed polarized and circularly left-handed polarized photons. The background contributions *I*_thermo_ are denoted by dashed lines as described in Methods. Experimental parameters: 75-nm-thin Bi_2_Se_3_ film, *E*_photon_=1.53 eV, *P*_laser_=20 mW and *T*_bath_=295 K.

Figure 1c shows an image of a Bi_2_Se_3_ film with two gold striplines serving as electronic contacts. We scan the pump laser across such a Bi_2_Se_3_ film and record the time-integrated photocurrent *I*_photo_ as a function of the laser position (Fig. 2a). At the metal interfaces (triangles), a photothermoelectric current is generated because of a laser-induced heat gradient and the large thermoelectric power of Bi_2_Se_3_. In between the two striplines, the time-integrated photocurrent averages to be close to zero (circles in Fig. 2a,b). Changing the polarization of the exciting laser from circularly left-handed to horizontally linear and then to circularly right-handed (Fig. 2c), we observe the sinusoidal fingerprint^15^ of a circular photogalvanic current with an amplitude *C* (Methods and Supplementary Fig. 1). The sinusoidal fits consider the circular and linear photogalvanic currents depending on the oblique angle *θ*=+17° (Supplementary Note 1 and Supplementary Fig. 1). The different background amplitudes of *I*_photo_ are highlighted as dashed lines in Fig. 2c. They represent the spatially varying, dominant photothermoelectric current *I*_thermo_. We introduce a fidelity *f*=*C/*(*C*+*I*_thermo_), which describes the ability to resolve the circular photogalvanic current with respect to *I*_thermo_. For all samples, we find *f* to be smaller than 40% in time-integrated measurements. The maximum value is achieved for the laser being positioned at the centre of the Bi_2_Se_3_ film (for example, circles in Fig. 2), where the overall *I*_thermo_ generated within the laser spot averages out towards the noise amplitude (*I*_thermo_→*A*_noise_~40 pA). As we show below, high-speed, time-resolved measurements provide a fidelity close to unity. This high fidelity can be achieved because photocurrent contributions on different timescales with different directions do not average out.

### Photothermoelectric currents at the contacts

**Figure 3:**
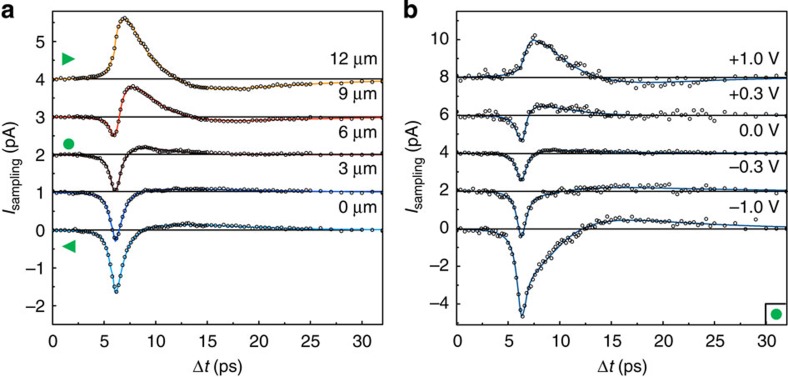
Time-resolved expansion dynamics of hot electrons. (**a**) *I*_sampling_ for excitation positions across a Bi_2_Se_3_ film in steps of 3 μm. The leftward (rightward) triangle marks an excitation position at the left (right) metal contact. The circle highlights an excitation at the centre of the Bi_2_Se_3_ film. Solid lines are fits to the data. Data are offset for clarity. Experimental parameters: 150-nm-thin Bi_2_Se_3_ film, *E*_photon_=1.59 eV, *P*_laser_=1 mW, *V*_sd_=0 V, *T*_bath_=77 K and a linear polarization. (**b**) *I*_sampling_ for excitation position in the centre of the Bi_2_Se_3_ film for −1 V≤*V*_sd_≤+1 V.

We measure the time-resolved photocurrent *I*_sampling_ for different excitation positions on a Bi_2_Se_3_ film from the right to the left contact (right- and leftward triangles in Fig. 3a). For all positions, we observe that *I*_sampling_ changes sign and therefore direction at a certain Δ*t*. This means that for short (long) times, a current with a direction towards the closer (farther) contact dominates. The origin of the opposing currents can be understood as follows. Within the first picosecond after excitation, the electrons thermalize to form a hot carrier ensemble^27^,^28^. They propagate away from the laser spot due to sample-internal potentials such as the thermopower and the density gradient. Close to the metal interfaces, the thermopower generated between Bi_2_Se_3_ and Au gives rise to the photothermoelectric current.

### Space and voltage dependence of the time-resolved photocurrents

In a time-of-flight analysis, we determine the ultrafast transport currents with opposing directions for each laser position (Supplementary Figs 2 and 3). The analysis allows us to estimate the fastest time-of-flight velocity of photogenerated hot electrons to be *v*_e_=(5.7±1.5) × 10^5^ ms^−1^ at room temperature (Supplementary Note 2), which agrees remarkably well with the group velocity of the Bi_2_Se_3_ at the given Fermi-energy^29^,^30^, as discussed below. In addition, we determine the decay time of the photocurrent signals to be (3.0±0.5) ps at room temperature, which is in agreement with the picosecond relaxation dynamics of hot electrons^26^,^27^.

In a next step, we focus the laser onto the centre of the Bi_2_Se_3_ film (Fig. 3b) where the time-integrated photocurrent is close to zero (for example, circle in Fig. 2a). At high bias, the data mimic the curves measured at zero bias at the right and left contacts (right- and leftward triangles in Fig. 3a). This means that at the centre in between the striplines (Fig. 3b), an externally applied *V*_sd_ gives rise to an additional drift of the photogenerated hot electrons. The polarity of the applied bias allows us to confirm that the dominating contribution stems from electrons and not holes (Supplementary Note 2 and Supplementary Fig. 4). The similarity of the top and bottom traces in Fig. 3a,b suggests that for certain values of *V*_sd_ at the centre, the corresponding electrostatic potential has an equivalent impact on the hot electron dynamics as the thermopower at the metal interfaces. In turn, at the centre of the sample and for *V*_sd_=0 V, the net field due to sample-internal potentials is zero. Particularly, the temporal tails of *I*_sampling_ are close to zero for Δ*t*≥10 ps at zero bias. We note that there is always an ultrafast photocurrent within the first picoseconds at this position (Fig. 3b).

### Helicity-dependent ultrafast photocurrents

**Figure 4:**
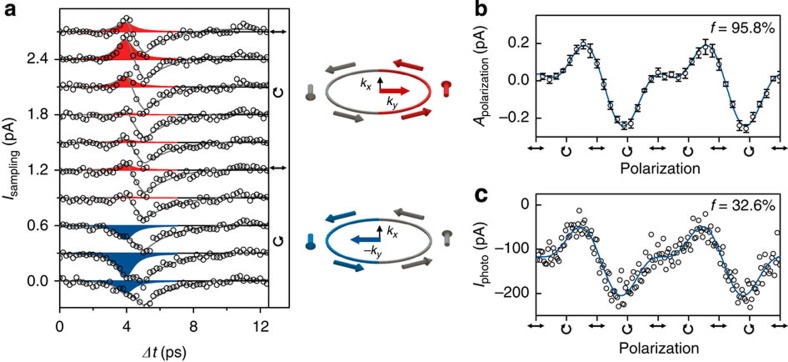
Polarization control of ultrafast currents. (**a**) *I*_sampling_ for excitation position close to the centre of a Bi_2_Se_3_ film for varying polarization. The laser is focused at a position at the centre of the Bi_2_Se_3_ film where the time-integrated photocurrent *I*_photo_ is close to zero. Solid lines are fits to the data. Data are offset for clarity. Red and blue peaks are polarization-controlled ultrafast currents in the direction of *k*_*y*_ to the right contact (red) or -*k*_*y*_ to the left contact (blue) with an in-plane spin polarization (middle panel). (**b**) Fitted amplitude of *I*_sampling_ for Δ*t*=4 ps in (**a**) versus photon polarization. Error bars are fitting errors. (**c**) Simultaneously measured time-averaged *I*_photo_ versus photon polarization. Experimental parameters: 90-nm-thin Bi_2_Se_3_ film, *E*_photon_=1.53 eV, *P*_laser_=20 mW and *T*_bath_=295 K.

Peculiarly, the sign and amplitude of the prevailing ultrafast contribution are completely controlled by the polarization of the exciting photons and their oblique angle of incidence. Figure 4a shows *I*_sampling_(Δ*t*) measured close to the centre of another Bi_2_Se_3_ film for a varying photon polarization. The fitting curves consider ultrafast transport currents with changing directions depending on the photon polarization (Supplementary Fig. 2). Particularly, for Δ*t*≈4 ps, the current amplitude depends only on the photon polarization. Microscopically, the helicity-dependent contribution results from an asymmetric excitation in *k*-space of the spin-polarized surface states caused by the peculiar helical symmetry of the surface states (Supplementary Fig. 1). The panels in Fig. 4a sketch the direction of the helicity-dependent current for circularly left-handed and right-handed polarized light (red and blue). The corresponding, measured peak amplitude follows the sinusoidal fingerprint of the circular and linear photogalvanic effects (Fig. 4b) with a fidelity *f* exceeding 95% for all temperatures. At the same excitation position, when the polarization dependence is measured in a time-integrated manner (Fig. 4c), the time-averaged background reduces *f* to be 32.6%, because the photocurrent contributions with different directions compete on long timescales.

## Discussion

It is insightful to highlight the dynamics of the photocurrents in Fig. 4a again. After the spin depolarization and intraband cooling (Δ*t*≥5 ps), the spin information and helicity protection are predominantly lost. Then, the expansion dynamics of the same (hot) electrons dominates the transport dynamics. These currents follow the combined influence of the thermopower and the electrostatic potentials. In the following, we will derive further insights into the interplay of the two potentials. Starting point is the fact that the optoelectronic expansion dynamics are dominated by photogenerated hot electrons that propagate at *v*_e_=(5.7±1.5) × 10^5^ ms^−1^ (Supplementary Note 2). Generally, the dispersion of the surface states can be written as^31^


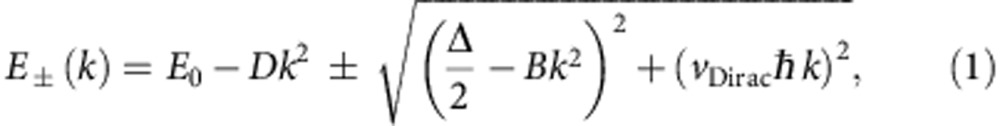


with *ℏk* the in-plane crystal momentum, *v*_Dirac_ is group velocity close to the Dirac point, *D*=−12.4 eV Å^2^ a quadratic term resulting from the broken particle-hole symmetry^32^, *B*=0 eV Å^2^ describing massive states^32^ and Δ the energy gap for the inter-surface coupling, which is zero for the investigated samples. The value for the binding energy of the Dirac point *E*_0_=(0.55±0.05) eV is determined from Hall-measurements (assuming an effective mass of 0.13·*m*_e_)^33^. Recent photoemission experiments on n-type Bi_2_Se_3_ and related topological materials demonstrate that, only a few 100 fs after photoexcitation, the electron distribution is already centred at the Fermi-energy^25^,^27^,^28^. From equation (1), we derive the group velocity 
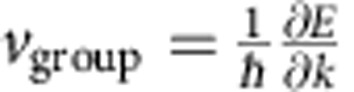
 to be (5.5±0.2) × 10^5^ ms^−1^ at the Fermi-energy *E*_Fermi_. We note that for bulk states, the principal order of the group velocity is in agreement with this value at the given Fermi-energy^32^. Importantly, the experimental value of *v*_e_ agrees remarkably well with the derived value and with the reported one determined from photoemission experiments^29^,^30^. All values exceed the saturation drift velocity as measured in Bi_2_Se_3_-based transistor devices^34^. In other words, the group velocity of photogenerated charge carriers determines the ultimate speed of the optoelectronic response in topological insulators.

Experimentally, this manifests itself in a linear time-of-flight diagram with *v*_e_=(5.7±1.5) × 10^5^ ms^−1^ (Supplementary Figs 2 and 3). To further discuss this surprising result, we use a two-temperature model *T*_e_ and *T*_phonon_ for the electron and phonon baths^35^,^36^. The electron heat capacity is well approximated by a linear temperature dependence *C*_e_~*C*′_e_*T*_e_, if *T*_e_≪*T*_Fermi_ with *T*_Fermi_=3,320 K the Fermi-temperature of the measured n-type Bi_2_Se_3_ films^37^. Within this model, we calculate the maximum electron temperature *T*_e_^max^=1,500 K for the experimental parameters as in Fig. 3a (ref. 35). The electron–electron collision time can be estimated to be *τ*_ee_~*ℏE*_Fermi_*/*(*k*_B_*T*_e_)^2^=9 fs, with *k*_B_ the Boltzmann constant^37^, and the electron-phonon scattering time is on the order of *τ*_e-phonon_~*ℏ/*(*k*_B_*T*_bath_)=94 fs. The latter was recently determined to be ~0.7 ps for optical phonons and ~2.3 ps for acoustic phonons^24^,^27^. In this regime (*τ*_ee_≪*τ*_e-phonon_), there exists a strong electron-lattice non-equilibrium, and the electron relaxation is governed by the electron–electron collisions^35^. It was reported that, hereby, the electronic heat transport occurs at the Fermi velocity after an ultrafast optical excitation of the electron bath^36^. The dynamics can be analytically described by a two-temperature model in the limit of a strong electron-lattice non-equilibrium in combination with an electron heat conductance *κ*_e_∝(*T*_e_)^−1^ (ref. 35). In the present experiment on Bi_2_Se_3_ films, the ultrafast current of hot electrons is measured. In our interpretation, this current carries the electronic heat to the contacts, and the corresponding heat transport at the Fermi velocity explains our data with linear time-of-flight diagrams (Supplementary Figs 2 and 3).

Our on-chip, time-domain THz spectroscopy allows us to reveal the impact of an electrostatic potential on such non-equilibrium expansion dynamics (Fig. 3b). Along this line, we estimate the maximum gradient of the local electron temperature profile to be ∇*T*_e_^max^~*T*_e_^max^*/*(0.5*d*_stripline_)=1,500 K*/*(0.5 × 15 μm)=200 K μm^−1^, with *d*_stripline_ the distance between the two striplines. In first approximation, the measured expansion dynamics of the hot electrons are identical for exciting the Bi_2_Se_3_ films at the contacts (top and bottom traces in Fig. 3a) and for an excitation spot in the centre of the sample at finite bias (top and bottom traces in Fig. 3b). For the results in Fig. 3b, the laser position is carefully chosen such that influence of the thermopower induced at the contacts is close to zero. Then for a finite bias *V*_sd_, the electrostatic potential gradient ∇*V*_electrostatic_ at the laser spot has an equivalent impact on the hot electron dynamics as the thermopower at the metal interfaces. We describe this thermopower at very short timescales as the product of *S*_Bi2Se3_·∇*T*_e_, with *S*_Bi2Se3_ a non-equilibrium, effective Seebeck coefficient. Comparing the amplitudes of the top and bottom traces of Fig. 3a,b, we extract |∇*V*_electrostatic_|=(820±440) V m^−1^ (Methods). In turn, we can estimate the effective Seebeck coefficient to be in the order of *S*_Bi2Se3_^minimum^~∇*V*_electrostatic_*/*∇*T*_e_^max^=−(4.1±2.2) μV K^−1^. The extracted value phenomenologically describes the non-equilibrium thermopower at the Bi_2_Se_3_-metal contacts. To the best of our knowledge, the above derivation is the first estimate of an effective Seebeck coefficient of non-equilibrium hot electron ensembles after a pulsed laser excitation. The derived non-equilibrium Seebeck coefficient is smaller than the typical quasi-equilibrium value *S*~−50 μV K^−1^ (ref. 38). This can be understood as follows: for the experimental parameters in Fig. 3, we concurrently find a time-averaged amplitude |*I*_photo_|=370 pA at the Bi_2_Se_3_-metal contact and at an acquisition time of ~ms. At this long timescale, the heat transport is governed by phonons. Accordingly, we numerically calculate a temperature increase at a Bi_2_Se_3_-metal contact of Δ*T*=47 mK (for both the phonon and electron baths). In turn, we derive a quasi-static Seebeck coefficient by the following expression Δ*V*~*S*_Bi2Se3_^quasistatic^ ·Δ*T*, with *S*_Bi2Se3_^quasistatic^=−23 μV K^−1^. For the sample and experimental conditions as in Fig. 2, we calculate *S*_Bi2Se3_^quasistatic^=−62 μV K^−1^ at room temperature. Both values are consistent with the above quasi-equilibrium value of the Seebeck coefficient. In other words, at long timescales, the heat transport is dominated by phonons. However, at ultrashort timescales, it is governed by a highly non-equilibrium expansion of hot electrons. In our interpretation, this non-equilibrium current of hot electrons carries the electronic heat to the contacts as long as there exists a strong electron-lattice non-equilibrium as discussed above.

Intriguingly, despite of such high-speed dynamics of the hot electrons, the data at Δ*t*~4 ps in Fig. 4a demonstrate that the helicity-dependent currents can be still addressed and read-out. The underlying physical reason is the protection of the helical states within the spin depolarization time. Only for Δ*t*≥5 ps in Fig. 4a, all currents have the same negative sign. That means that, for this particular experiment, still a tiny thermopower potential drives these currents to the left contact, which is consistent with the negative offset observed in the time-integrated measurement (Fig. 4c). Again, for Δ*t*≥5 ps, there is no polarization control for these currents anymore, since the pump-laser pulse is off and the spin depolarization has occurred (Fig. 4a).

The circular and linear photogalvanic effects are induced via surface states and not bulk states because of the broken inversion symmetry at the surface of Bi_2_Se_3_ (refs 15, 17). A normal incidence geometry (*θ*=0°) suppresses the photogalvanic effects (Supplementary Fig. 1), indicating an in-plane spin distribution and more fundamentally, an in-plane rotational symmetry of the involved electron states in Bi_2_Se_3_. Generally, there may be a small contribution from a helicity-independent transverse photon drag effect^17^. Contributions from Rashba-split bulk states at a surface inversion layer can be assumed to be negligible for the examined range of *E*_photon_ (Supplementary Note 3 and Supplementary Fig. 5). Overall, the ultrafast polarization-controlled photocurrent is limited by the spin lifetime in Bi_2_Se_3_.

To conclude, our experiments demonstrate the temporal separation of helicity-dependent surface photocurrents from polarization-independent bulk currents even at room temperature, and they reveal the onset of photothermoelectric currents at ultrafast timescales. We elaborate the connection between the non-equilibrium currents of hot electrons directly after the pulsed laser excitation and the time-averaged thermoelectric currents, which are typically described in the framework of a Seebeck coefficient. A time-of-flight analysis yields a speed of the photogenerated electrons that is consistent with the group velocity at the Fermi-energy of the Bi_2_Se_3_. Our results significantly advance the understanding of the optical excitation scheme and the ultrafast photocurrent dynamics in topological insulators. The time-delayed photothermoelectric currents in Bi_2_Se_3_ are partly caused by the n-doping of the material. They can be reduced by utilizing topological insulators with a Fermi-energy in the Dirac cone, such as Bi_2_Te_2_Se or (Bi_1-x_Sb_x_)_2_Te_3_ (refs 7, 8). The picosecond response time of the surface currents proves the anticipated potential of topological insulators as promising materials for high-speed optoelectronic applications from the THz to the infrared range.

## Methods

### Fabrication of the Bi_2_Se_3_ films

The investigated Bi_2_Se_3_ films have a thickness of 50 nm≤250 nm and a lateral dimension of >15 μm. We produce the Bi_2_Se_3_ films from 99.999% pure crystalline Bi_2_Se_3_ granulate via exfoliation. We analyse the homogeneity and the dimensions of the films using optical microscopy, atomic force microscopy and white light interferometry. The presented results have been reproduced on three independent samples, which have a thickness of 75, 90 and 150 nm. Hall-measurements on a 65-nm-thin Bi_2_Se_3_ film from the same batch yield an electron density of ~4·10^19^ cm^−3^ at room temperature.

### Design of the stripline circuit

Starting point is a 430-μm-thick sapphire substrate covered with a 300-nm-thin layer of ion-implanted silicon (Si). In a first optical lithography step, the Auston-switch geometry is formed via HF etching. The remaining silicon strip serves as a field probe (Auston-switch). Bi_2_Se_3_ films are mechanically transferred onto the substrate by exfoliation. In a second optical lithography step, we evaporate 10 nm titanium (Ti) and 110–200 nm gold (Au) to form the waveguide circuits contacting the Bi_2_Se_3_ films and the read-out of the Auston-switch. The distance between two parallel striplines is 15 μm. Each stripline itself has an individual width of 5 μm. The Bi_2_Se_3_ films are placed at a typical distance of 100 μm≤800 μm from the Auston-switch. The bias voltage *V*_sd_ is applied between striplines (see Fig. 1). The striplines including the Bi_2_Se_3_ films have a two-terminal resistance of 2.9 kΩ, while the Bi_2_Se_3_ films have a resistance of ~55 Ω. Hereby, the gradient ∇*V*_electrostatic_ can be estimated from the bias voltage via ∇*V*_electrostatic_=*V*_sd_*/*15 μm · 55 Ω/2.9 kΩ.

### Time-integrated photocurrent spectroscopy

The time-averaged *I*_photo_ is measured in between the two striplines (Fig. 1b) with a current-voltage amplifier.

### On chip time-domain THz photocurrent spectroscopy

The Bi_2_Se_3_ films in the stripline circuit are optically excited by a pump pulse with ~200 fs pulse length generated by a titanium:sapphire laser at a repetition frequency of ~76 MHz with a photon energy of *E*_photon_=1.53 eV and *E*_photon_=1.59 eV. After excitation, an electromagnetic pulse starts to travel along the striplines. A field probe senses the transient electric field of the travelling pulse (Fig. 1b). Here we utilize an Auston-switch based on ion-implanted silicon. At a time delay Δ*t* with respect to the pump pulse, the Auston-switch is short-circuit by a probe pulse for the duration of the lifetime of the photogenerated charge carriers in the silicon (*τ*≤ 1 ps). During this time period, the transient electric field present at the field probe drives the current *I*_sampling_. In turn, measuring *I*_sampling_(Δ*t*) yields information on the optoelectronic response of the Bi_2_Se_3_ with a picosecond time-resolution. The time a THz-pulse travels from the Bi_2_Se_3_ film to the field probe can be estimated via *t*_travel_=*d*· *n*_sapphire_/*c*=0.5 mm · 3.07/*c*~5.1 ps for a distance of 0.5 mm between the Bi_2_Se_3_ film and the Auston-switch. The striplines have a total length exceeding 48 mm. Thus, reflections at the end of the striplines are expected for Δ*t*≥490 ps. These reflections are strongly reduced due to damping and radiation losses. Therefore, no reflections overlap with the electromagnetic signal coming directly from the Bi_2_Se_3_ films in our data. The data of Figs 2 and 4 are presented for room temperature. The time-of-flight data in Fig. 3 are depicted for 77 K, because the transfer characteristics of the striplines and therefore the signal-to-noise ratio are enhanced at lower temperatures. A time-of-flight analysis of data at room temperature is presented in the Supplementary Fig. 3. The position of the pump-spot is set with a spatial resolution of ~100 nm, while the position of the probe-spot is kept constant throughout the experiments. All measurements of *I*_sampling_ were carried out utilizing an optical chopper system, a current-voltage converter connected to the field probe and a lock-in amplifier. The spot size (FWHM) of the pump-laser is 3–4 μm for all time-resolved experiments and up to 9 μm for the time-integrated experiments. The laser power of the pump (probe) pulse is chosen to be in the range of 0.1–20 mW (80–150 mW). All data are taken in vacuum (~10^−5^ mbar) to prevent the effect of photo-desorption of oxygen on the surface of the Bi_2_Se_3_. The measurements have been reproduced for different temperatures between 4 and 295 K.

### Polarization control and sinusoidal fitting function

The polarization-dependent photocurrents are characterized by measuring *I*_photo_ or *I*_sampling_, while rotating a *λ*/4-waveplate by an angle *φ*. The rotation changes the photon polarization with a period of 180° from linearly polarized (*φ*=0°) to left-handed circular (*φ*=45°), to linearly (*φ*=90°), to right-handed circular (*φ*=135°) and to linearly (*φ*=180°). The fitting curves in Figs 2c and 4b,c describe the photocurrent in the *y*-direction of the Bi_2_Se_3_ films (Fig. 1b) as *j*(*φ*)=C sin 2*φ*+*L*_1_ sin 4*φ*+*L*_2_ cos 4*φ*+*D*. As recently demonstrated^15^, *C* describes the helicity-dependent circular photogalvanic effect with a rotational in-plane symmetry in Bi_2_Se_3_. *L*_1_ comprises the helicity-independent linear photogalvanic effect. *L*_2_ and *D* are bulk contributions (Supplementary Note 1).

## Author contributions

C. Kastl and C. Karnetzky performed the experiments and analysed the data together with A.W.H. A.W.H., C. Kastl and C. Karnetzky conceived the study and co-wrote the paper with H.K.

## Additional information

**How to cite this article:** Kastl, C. *et al*. Ultrafast helicity control of surface currents in topological insulators with near-unity fidelity. *Nat. Commun.* 6:6617 doi: 10.1038/ncomms7617 (2015).

## Supplementary Material

Supplementary Information Supplementary Figures 1-5, Supplementary Notes 1-3, and Supplementary References
