# Supplementary material for: Ultrafast helicity control of surface currents in topological insulators with near-unity fidelity
Source: Nat Commun. 2015 Mar 26;6:6617. doi: 10.1038/ncomms7617 (PMC4389261; doi:10.1038/ncomms7617)
Supplement: Supplementary Information — Supplementary Figures 1-5, Supplementary Notes 1-3, and Supplementary References [file ncomms7617-s1.pdf]

Supplementary Figure 1

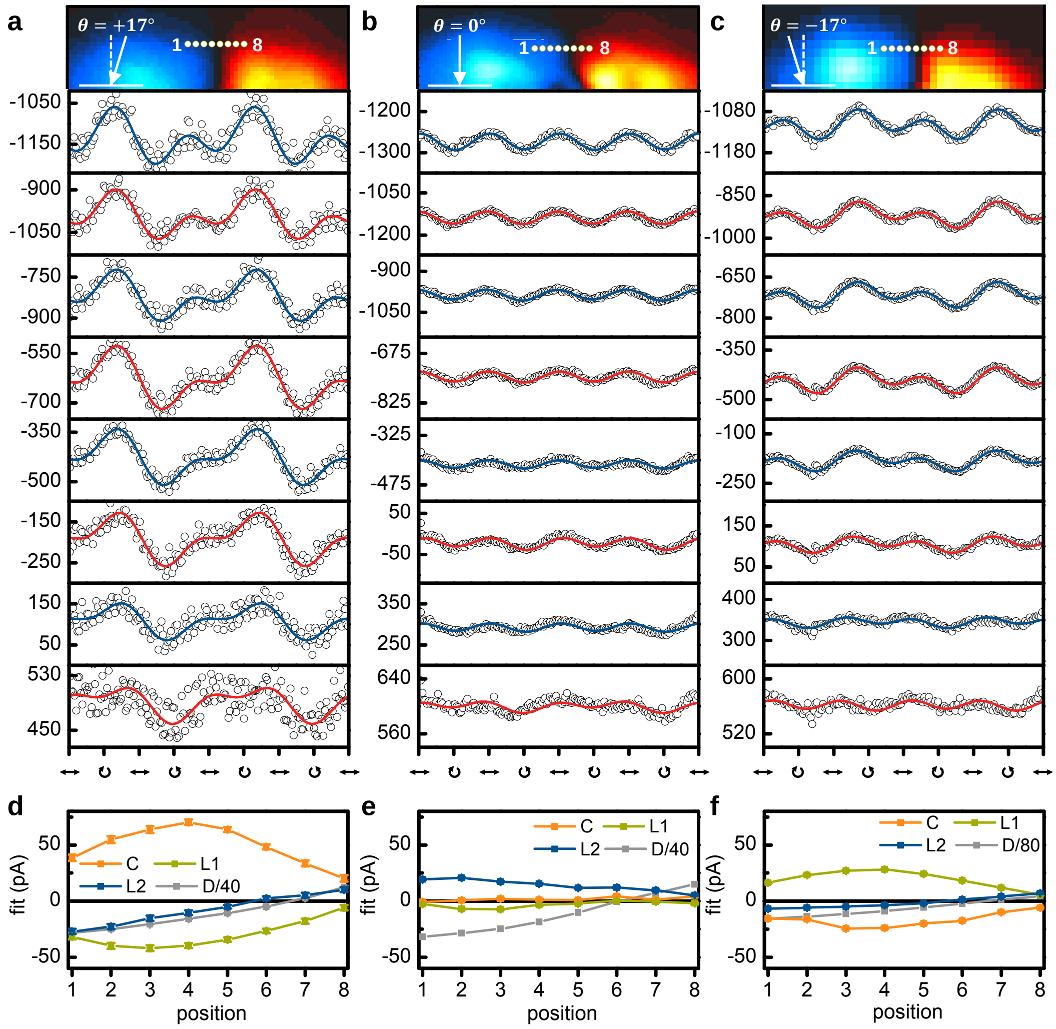

Supplementary Figure 1 Polarization dependence of the photocurrent I_{photo} for a varying oblique angle θ. (a) (b) and **(c)** θ = -17°, 0° and +17° as defined in the top panels. The top panels display photocurrent maps. The dots in the maps define the eight positions 1, … to 8, at which the polarization dependence of I_{photo} are measured. The eight polarization dependences of I_{photo} are depicted below the photocurrent maps, respectively. The polarization changes from linear to circularly left-handed to circularly right-handed. Experimental parameters: 90 nm thin Bi_2Se_3-film, E_{photon} = 1.53 eV, P_{laser} = 10 mW, and T_{bath} = 295 K. Lines are sinusoidal fits according to $j(\phi) = C \sin 2\phi + L_1 \sin 4\phi + L_2 \cos 4\phi + D$. The parameter C describes the helicity-dependent circular photogalvanic effect with a rotational in-plane symmetry in Bi_2Se_3. L_1 comprises the helicity-independent linear photogalvanic effect. L_2 and D are bulk contributions. **(d) (e)** and **(f)** depict the corresponding fitting parameters. Most importantly, C, L_1 change their current polarity for θ = -17° and +17°, and they are close zero for θ = 0°. For clarity, D is scaled down as specified.

Supplementary Figure 2

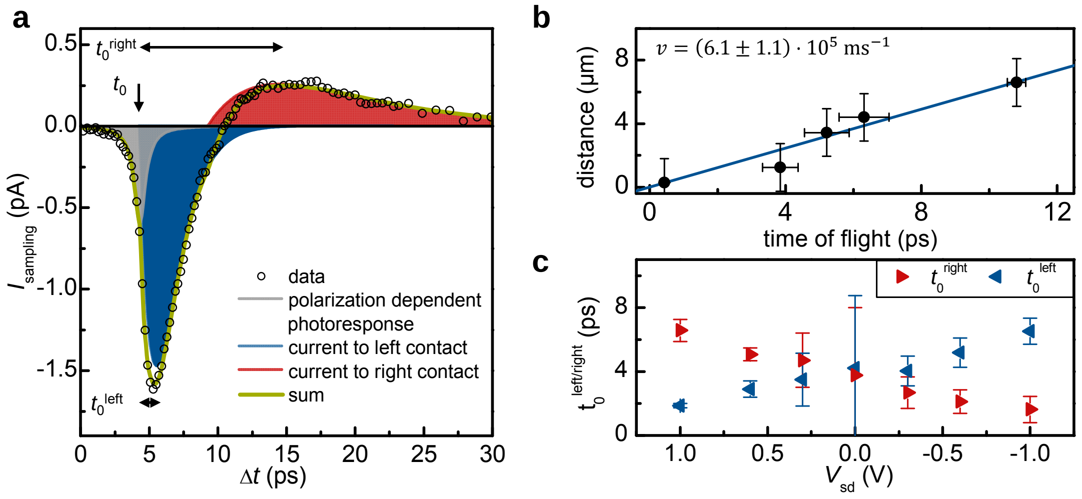

Supplementary Figure 2 Time-of-flight analysis of photogenerated hot carriers the Bi_2Se_3-film with thickness of 150 nm at 77 K. (a) $I_{sampling}$ as a function of the time delay Δt. This graph introduces the fitting functions and the time-delays at which the current to the right contact (t_0^{right}) and the one to the left contact (t_0^{left}) currents are maximum. The time delays t_0^{right} and t_0^{left} are determined with respect to t_0. **(b)** Distance of propagation vs. time of flight of the photogenerated electrons. Errors bars include fitting errors (error of time axis) and position uncertainty due to the extended laser spot (error of distance axis). **(c)** Voltage dependence of t_0^{right} and t_0^{left} for $|V_{sd}| \leq$ 1 V at the middle of the Bi_2Se_3 film. Error bars are fitting errors. Experimental parameters: 150 nm thin Bi_2Se_3-film, E_{photon} = 1.59 eV, P_{laser} = 1 mW, and T_{bath} = 77 K.

Supplementary Figure 3

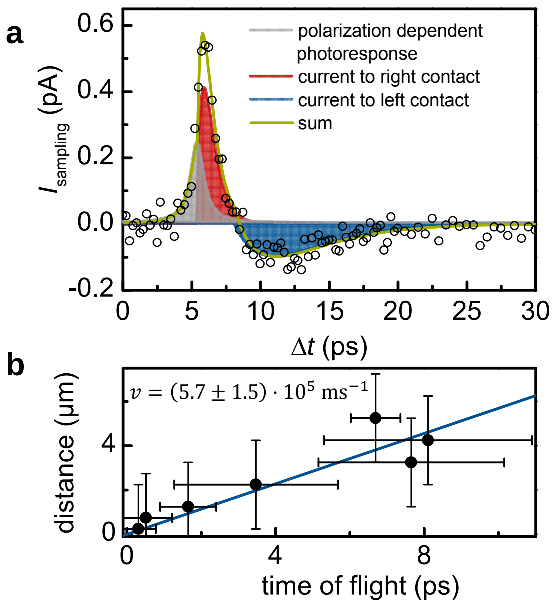

Supplementary Figure 3 Time-of-flight analysis of photogenerated hot carriers in the Bi_2Se_3-film with thickness of 90 nm at room temperature. (a) $I_{sampling}$ as a function of the time delay Δt with fitting functions. **(b)** Distance of propagation vs. time of flight of the photogenerated electrons. Errors bars include fitting errors (error of time axis) and position uncertainty due to the extended laser spot (error of distance axis). Experimental parameters: 90 nm thin Bi_2Se_3-film, E_{photon} = 1.53 eV, P_{laser} = 20 mW, and T_{bath} = 295 K.

Supplementary Figure 4

a

	Negative sign of ultrafast current	Positive sign of ultrafast current
electrons	large t_0^{left}<br>small t_0^{left}	large t_0^{right}<br>small t_0^{right}
holes	large t_0^{right}<br>small t_0^{right}	large t_0^{left}<br>small t_0^{left}

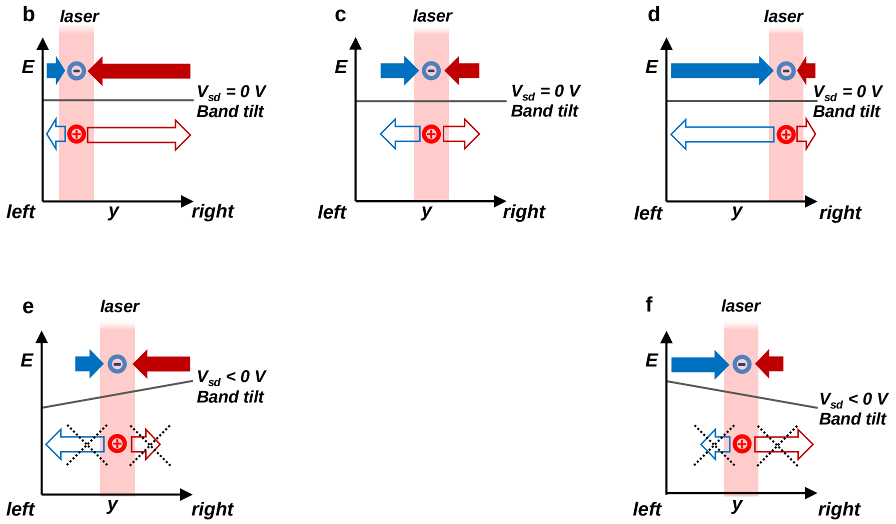

Supplementary Figure 4 Schematic of the expansion direction of photogenerated electrons and holes in the Bi_2Se_3-film. (a) Arrows show the currents of electrons and holes. The direction of the arrows indicates the current direction. The length indicates the time of flight towards the contact. **(b)** Currents for excitation at the left contact without external bias (flat band). Currents due to charge carriers propagating to the right are delayed compared to currents to the left. **(c)** For excitation at the middle all currents cancel out. **(d)** Currents for excitation at the right contact without external bias. **(e)** and **(f)** Currents for excitation at the left (right) contact with external bias applied. The electric field shifts the transport currents. Note the asymmetry between electrons and holes. The experimental condition in **b** and **e** yield equal time-resolved photoresponses. Equally, **d** and **f** yield similar results. Both consistencies are only possible, when the transport is dominated by electrons and not by holes, as indicated by the crossed-out contribution of the hole currents.

Supplementary Figure 5

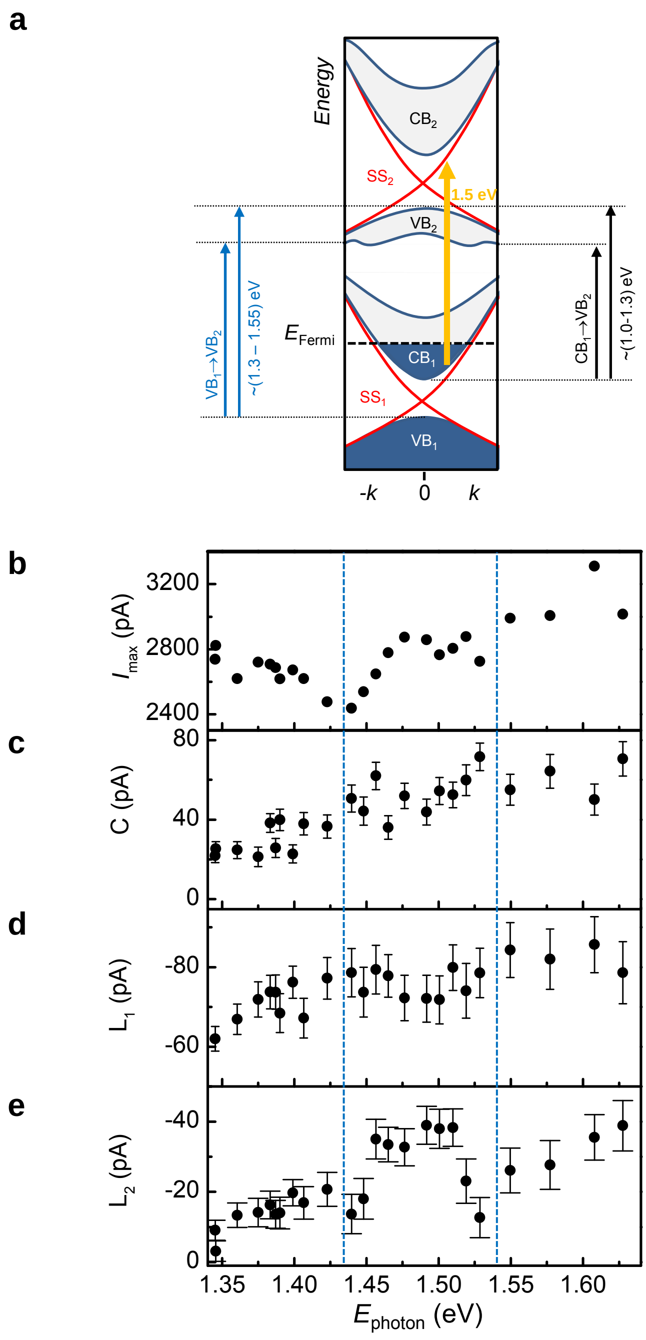

Supplementary Figure 5 Polarization dependent photocurrents vs. photon energy. (a) Sketch of the band diagram of Bi_2Se_3 based on ref. [30]. **(b)** Maximum of $|I_{photo}|$ vs. E_{photon}. **(c)** Circular photogalvanic effect described by parameter C vs. E_{photon}. **(d)** Linear photogalvanic effect described by parameter L_1 vs. E_{photon}. **(e)** Linear photogalvanic effect described by parameter L_2 vs. E_{photon}. Experimental parameters: 75 nm thin Bi_2Se_3-film, P_{laser} = 20 mW, and T_{bath} = 295 K.

Supplementary Note 1

Polarization dependent photocurrents in Bi_2Se_3. Microscopically, the circular photogalvanic effect can be understood by the following simplified symmetry argument. The pump laser excites the Bi_2Se_3-films under an oblique angle θ of incidence with a projection along the *x*-direction (Figure 1b of the main manuscript). The helicity of the vector potential of the photons changes sign under a symmetry transformation with a mirror axis along this *x*-direction[1][2][3]. Only a current along the *y*-direction but not along the *x*-direction can follow this sign change (considering the symmetry C_{3v} of the topological surface of Bi_2Se_3). In turn, the current of spin-polarized electrons to the contacts – as measured in our experiments – follows a sine curve as a function of the photon polarization[3]. We use this sinusoidal fingerprint to fit our data. In addition, the current is suppressed for a normal angle of incidence, as demonstrated below. The symmetry of the bulk states of Bi_2Se_3 is D^5_{3d}, which does not allow photogalvanic effects in the bulk[1][2][3].

Supplementary Figs. 1a, 1b, and 1c depict the polarization dependent photocurrents of a Bi_2Se_3 film under three angles $\theta = -17°$ (a), 0° (b), and +17° (c) of (oblique) incidence at room temperature. Fitting the dependences with the sinusoidal fit function as given in the Methods section, we can extract a position dependence of the parameters C, L_1, L_2 and D (Supplementary Figs. 1d, 1e, and 1f). The parameters C and L_1 have opposite sign, as previously reported[4], and they vary qualitatively similar as a function of position. Furthermore, C and L_1 switch polarity for interchanging θ from -17° to +17° (d) and (f). Most importantly, the parameters C and L_1 are largely reduced for $\theta = 0°$ (e). Based on these observations, we interpret C to originate from the helicity-dependent circular photogalvanic effect within surface states with an in-plane helicity. L_1 stems from the helicity-independent linear photogalvanic effect, and it may comprise small contributions from the transverse photon drag effect[1]. As discussed below in the section "Energy levels in nanofabricated Bi_2Se_3 films", we consistently observe that C and L_1 occur at the same photon energies and therefore, optical transition energies within the Bi_2Se_3-films. Furthermore, for $\theta \neq 0°$, we attribute the components D and L_2 to originate from transitions in the bulk states of Bi_2Se_3. The above interpretations are in agreement with an earlier report[4].

We note that the range of E_{photon} = 1.53 – 1.6 eV used for the essential results in the main manuscript is chosen such that the contribution of the bulk and Rashba states can be considered to be negligible (see Supplementary Note 3 "Energy levels in nanofabricated Bi_2Se_3 films").

Supplementary Note 2

Time-of-flight analysis of the photogenerated hot carriers. In the Supplementary Figure 2a, the bottom trace of Figure 3a of the main manuscript is reproduced (open circles). Assuming in a simplified model a time-scale separation of the different processes, we fit the data with the following function (green line):

$$I_{\mathrm{fit}}(\Delta t) = I_{\mathrm{polarization}}(\Delta t) + I_{\mathrm{right}}(\Delta t) + I_{\mathrm{left}}(\Delta t) \qquad \text{(Supplementary Equation 1).}$$

The first term $I_{\mathrm{polarization}}$ describes the polarization-dependent part of I_{sampling} which can be fitted by a Lorentzian with the following form:

$$I_{\mathrm{polarization}}(\Delta t) = A_{\mathrm{polarization}} \cdot \sigma / [\pi (\Delta t^2+\sigma^2)], \qquad \text{(Supplementary Equation 2)}$$

with $A_{\mathrm{polarization}}$ being the integrated area and σ the half-width at half-maximum (HWHM) of the Lorentzian peak (gray in the Supplementary Figure 2a). The Lorentzian considers phenomenologically the dispersion and attenuation of the electromagnetic transient running along the striplines before it is detected at the field probe. The two slower decaying components I_{right} and I_{left} have the form

$$I_{\mathrm{i}}(\Delta t) = A_{\mathrm{i}}/\delta_{\mathrm{i}}^2 \cdot \Delta t\, e^{-\Delta t/\delta_{\mathrm{i}}} \cdot \Theta(\Delta t), \ (i = \text{right, left}) \ \text{(Supplementary Equation 3)}$$

with A_{i} being the corresponding integrated area and δ_{i} the characteristic decay time of I_{i} (i = right, left), and $\Theta(t)$ the Heaviside step function. I_{right} describes the propagation of hot carriers to the right contact (red in the Supplementary Figure 2a), and I_{left} describes the propagation of hot carriers to the left contact (blue). We note that in Figs. 3b (zero bias) and in Figure 4a of the main manuscript, experiments are presented in which the contributions of $I_{\mathrm{right}}(\Delta t)$ and $I_{\mathrm{left}}(\Delta t)$ are nearly negligible. It can be clearly seen that the residual peak fitted by $I_{\mathrm{polarization}}(\Delta t)$ is a Lorentzian and not a Gaussian.

We observe that $I_{\mathrm{polarization}}$ is the only term in the time-resolved photocurrent I_{sampling} which depends on the photon polarization (Figure 4a of the main manuscript). In addition, this term vanishes on a time scale which is consistent with the spin-depolarization time in Bi_2Se_3 convoluted with the time-resolution of the presented experiment (~1ps)[4]. We point out that the striplines act as nearfield antennas in the THz-regime. Hereby, the striplines pick up the electromagnetic transients produced by the photocurrents quasi instantaneously via the speed of light[5]. In turn, the first peak term $I_{\mathrm{polarization}}$ defines the time t_0 when the pump laser excites the Bi_2Se_3-films. I_{right} and I_{left} describe slower processes of hot charge carries propagating within the Bi_2Se_3-films. Consistently, both I_{right} and I_{left} depend on the excitation position with respect to the Bi_2Se_3-films and on V_{sd} (Figure 3 of the main manuscript). We note that the simplifying fit of Supplementary Equation 1 partially does not comprise the situation when polarization-dependent and polarization-independent optoelectronic processes somewhat overlay. For instance, this can happen at the contacts (such as top trace of Figure 3a) and at the presence of electric fields (top trace of Figure 3b). That is the reason why all helicity-dependent currents are only discussed for the center of the Bi_2Se_3-films and for a bias close to zero (as in Figure 4).

With respect to t_0, the maxima of I_{right} (I_{left}) occur at a delayed time t_0^{right} (t_0^{left}) (Supplementary Figure 2a) depending on the distance to the contacts. The Supplementary Figure 2b represents a corresponding time-of-flight diagram of the 150 nm thin Bi_2Se_3-film at 77 K which allows us to estimate an upper limit of the propagation velocity of the fastest photogenerated electrons to be $v_e = (6.1 \pm 0.6) \times 10^5$ ms^{-1}. For the 90 nm thin Bi_2Se_3-film at 295 K (Supplementary Figure 3), we extract $v_e = (5.7 \pm 0.7) \times 10^5$ ms^{-1}. Both values are consistent with the group velocity of $(5.5 \pm 0.2) \times 10^5$ ms^{-1} as calculated in the section "Discussion" of the main manuscript. We point out that the maxima of I_{right} and I_{left} comprise the fastest propagating photogenerated electrons. The decaying tails of the I_{right} and I_{left} comprise slower photocurrent dynamics with a time scale of several picoseconds. The underlying processes are a combination of diffusion and drift of hot electrons due to the thermopower and electrostatic potentials. The latter scenario is proven by the fact that t_0^{right} and t_0^{left} depend on V_{sd} at the middle of the Bi_2Se_3-films (Supplementary Figure 2c).

The bias dependence further allows us to conclude that the time-resolved photocurrents are dominated by photogenerated electrons and not holes. This will be discussed in the following. In Supplementary Figure 4 we depict schematically the photocurrents of electrons and holes for different excitation positions and under different external bias conditions. The arrows show the currents (Supplementary Figure 4a), whereby the direction of the arrows indicates the current direction and the length indicates the time of flight towards the contact. In the Supplementary Figure 4b, we depict the currents for excitation at the left contact. The currents due to charge carriers propagating to the right are delayed compared to currents to the left, as indicated. The situation is reversed in Figure 4d. For excitation exactly at the middle (Supplementary Figure 4c) all currents are equally delayed and cancel out. In the Supplementary Figs. 4e and 4f, the electric field shifts the transport currents. Note the asymmetry between electrons and holes. Experimentally we find in Figure 3 of the main manuscript, that the experimental condition in the Supplementary Figs. 4b and 4e yield qualitatively equal time-resolved photoresponses. Equally, supplementary Figure 4d give the equivalent results as Figure 4f. Both consistencies are only possible, when the transport is dominated by electrons and not by holes, as indicated by the crossed-out contribution of the hole currents in the Supplementary Figs. 4e and 4f.

Supplementary Note 3

Energy levels in nanofabricated Bi_2Se_3 films. Supplementary Figure 5a sketches the band diagram for *n*-type Bi_2Se_3 without band bending effects at the surface, as was experimentally derived by two independent experiments using a two-photon angle resolved photoemission spectroscopy (2PPES)[10][11]. A very similar band diagram was theoretically computed by density functional theory[10][11][12]. Generally, the relative energy differences of the bulk bands and the surface states depend on the surface treatment of the Bi_2Se_3 films; i.e. the exposure to air as is essential for nanofabricated Bi_2Se_3 films with metal contacts. For all treatments, Bi_2Se_3 ends up to be *n*-type with a Fermi energy of several 100s of meV, and the transition energies can additionally vary by ~0.1 eV.[13][14][15] Importantly, the coexistence of the topological surface states to such a surface inversion layer was experimentally verified.[15][16]
We fabricated a Bi_2Se_3-film with a height of 65 nm from the same batch of materials with four metal contacts to perform Hall-measurements. We obtain an electron density of $\sim 4\cdot 10^{19}$ cm^{-3} and an electron mobility of 470 cm²/Vs at room temperature. Accordingly, we obtain a Fermi-energy of ~0.3 eV consistent with earlier reports. The depth of the inversion layer can be estimated via the Thomas-Fermi screening length to be ~6 nm, consistent with earlier reports[13][15][16].

To perform 2PPES on nanofabricated Bi_2Se_3 films is a nearly impossible task. However, the results by refs. [10][11][12] verify for *n*-type Bi_2Se_3 that a photon with energy of ~1.5 eV excites charge carriers from the first conduction band (CB_1) to an unoccupied, topological non-trivial Dirac cone of the surface state SS_2 (orange arrow and CB_1 and SS_2 as in the Supplementary Figure 5a). This energy is slightly less than the photon energy used in our optoelectronic experiments. Therefore, we exploit the photocurrent signal itself to conclude which surface states are involved in the polarization dependent currents.

Supplementary Figs. 5b – 5d depict the (polarization dependent) photocurrent I_{photo} vs. E_{photon}. In particular, for each E_{photon}, a photocurrent map is measured, and the corresponding maximum amplitude of $|I_{photo}|$ of the maps is extracted vs. E_{photon} (Supplementary Figure 5b). Then, at the center position of the maps, the polarization dependent photocurrent is measured and analyzed. Supplementary Figure 5b (c,d) depicts the corresponding parameter C (L_1, L_2) for a laser energy in the range of 1.33 eV $\leq E_{photon} \leq$ 1.66 eV. We find that the parameter C and L_1 show a very similar energy dependence throughout the examined range of E_{photon}. This corroborates the interpretation that they stem from related optoelectronic processes – the circular and the linear photogalvanic effects from surface states (see section "Polarization dependent photocurrents in Bi_2Se_3"). The correlation between C and L_1 has been experimentally verified by earlier work[4].

The dispersion of the states SS2 and CB1 are such that the transition $CB_1 \rightarrow SS_2$ has a maximum energy at a non-zero *k*-vector $\leq$ 0.1 Å^{-1} (orange arrow in the Supplementary Figure 5a)[10] and for all other *k*-vectors, the transition energy is smaller. For an *n*-type inversion layer with a Rashba spin-split CB1, the Rashba spin-split bands are ~0.13 eV above a non-split CB1 [13][15]. Therefore, the possible

transition energy $CB_1 \rightarrow SS_2$ at the surface is reduced by about ~0.1 eV. In turn, we can assume for a photon energy $E_{photon} \geq$ ~1.5 eV, the transition $CB_1 \rightarrow SS_2$ is not relevant at the surface. In the bulk, the transition cannot occur either because there, the surface states do not exist.

We note that L_2 exhibits a maximum at 1.45 eV $\leq E_{photon} \leq$ 1.53 eV (supplementary Figure 5e), which we attribute to a dominant transition $VB_1 \rightarrow VB_2$ in this energy range (supplementary Figure 5a). Hereby, we attribute L_2 to originate from bulk transitions in Bi_2Se_3. This is again in agreement with conclusions based on earlier optoelectronic experiments[4]. Consistently, the maximum photocurrent $|I_{photo}|$ as depicted in the supplementary Figure 5a varies in a similar manner as L_2 for 1.45 eV $\leq E_{photon} \leq$ 1.53 eV. As recently demonstrated[13], the valence band VB_1 at the surface is not spin-split. Therefore, the transition $VB_1 \rightarrow SS_2$ does not contribute to the circular photogalvanic effect. Last but not least, the bulk transition $CB_1 \rightarrow CB_2$ can only be expected for a photon energy $E_{photon} \geq$ 1.7 eV [10][12].

To summarize, we chose E_{photon} = 1.53 eV for the presented time-resolved, polarization-dependent experiments (Figure 4a in main manusscript), because L_2 exhibits a minimum, and C and L_1 are in a maximum range. Based on the above arguments, we optically excite the transitions $SS_1 \rightarrow SS_2$. This interpretation is consistent with the fact that we find a propagation velocity of $(5.5 \pm 0.2) \times 10^5$ ms^{-1} at room temperature (see Supplementary Note 2 “Time-of-flight analysis of the photogenerated hot carriers”).

We point out that the chosen range of E_{photon} is in the range 1.5 eV < $E_{photon} \leq$ 1.7 eV, in which we can argue that the transition $SS_1 \rightarrow SS_2$ is responsible for the circular photogalvanic effect. However, the revealed ultrafast optoelectronic dynamics of surface states in topological insulators applies throughout the whole vis-NIR range, since transition $SS_1 \rightarrow SS_2$ is always excited in this range.

Supplementary References

[1] Ganichev, S. D. & Prettl, W. Spin photocurrents in quantum wells. *J. Phys. Condens. Matter* **15,** R935–R983 (2003).

[2] Hosur, P. Circular photogalvanic effect on topological insulator surfaces: Berry-curvature-dependent response. *Phys. Rev. B* **83,** 035309 (2011).

[3] Junck, A., Refael, G. & Von Oppen, F. Photocurrent response of topological insulator surface states. *Phys. Rev. B* **88,** 075144 (2013).

[4] McIver, J. W., Hsieh, D., Steinberg, H., Jarillo-Herrero, P. & Gedik, N. Control over topological insulator photocurrents with light polarization. *Nat. Nanotechnol.* **7,** 96–100 (2012).

[5] Prechtel, L. *et al.* Time-resolved ultrafast photocurrents and terahertz generation in freely suspended graphene. *Nat. Commun.* **3,** 646 (2012).

[6] Brorson, S., Fujimoto, J., & Ippen, E. Femtosecond electronic heat-transport dynamics in thin gold films. *Phys. Rev. Lett.* **59**, 1962–1965 (1987).

[7] Kanavin, A., Smetanin, I., & Isakov, V. Heat transport in metals irradiated by ultrashort laser pulses. *Phys. Rev. B* **57**, 698–703 (1998).

[8] Wang, Y. *et al.* Measurement of Intrinsic Dirac Fermion Cooling on the Surface of the Topological Insulator Bi2Se3 Using Time-Resolved and Angle-Resolved Photoemission Spectroscopy. *Phys. Rev. Lett.* **109**, 127401 (2012).

[9] Crepaldi, A. *et al.* Ultrafast photodoping and effective Fermi-Dirac distribution of the Dirac particles in Bi_2Se_3. *Phys. Rev. B* **86,** 205133 (2012).

[10] Sobota, J. A. *et al.* Direct optical coupling to an unoccupied Dirac surface state in the topological insulator Bi_2Se_3. *Phys. Rev. Lett.* **111,** 136802 (2013).

[11] Niesner, D. *et al.* Unoccupied topological states on bismuth chalcogenides. *Phys. Rev. B* **86,** 205403 (2012).

[12] Eremeev, S. V., Silkin, I. V., Menshchikova, T. V., Protogenov, A. P. & Chulkov, E. V. New topological surface state in layered topological insulators: Unoccupied dirac cone. *JETP Lett.* **96,** 780–784 (2013).

[13] Benia, H. M., Lin, C., Kern, K. & Ast, C. R. Reactive chemical doping of the Bi_2Se_3 topological insulator. *Phys. Rev. Lett.* **107,** 177602 (2011).

[14] Bahramy, M. S. *et al.* Emergent quantum confinement at topological insulator surfaces. *Nature Comm.* **3**, 1159 (2012).

[15] Benia, H. M., *et al.* Origin of Rashba splitting in the quantized subbands at the Bi_2Se_3 surface. *Phys. Rev. B* **88**, 081103 (R) (2013).

[16] Bianchi, M. *et al.* Coexistence of the topological state and a two-dimensional electron gas on the surface of Bi_2Se_3. *Nature Comm.* **1**, 128 (2010).
